# Oral health knowledge, attitude and behaviour of dental students in a private university

**DOI:** 10.1038/s41405-019-0024-x

**Published:** 2019-10-24

**Authors:** Azhar Abdulrahman Al-wesabi, Fatma Abdelgawad, Hisako Sasahara, Kamal El Motayam

**Affiliations:** 1 Paediatric Dentistry and Dental Public Health Department, Faculty of Dentistry, Cairo University, 11 El-Saraya Street, El Manial, Cairo, Egypt; 2 Integrated Health, Institute of Biomedical and Health Sciences Hiroshima University, Hiroshima, Japan

**Keywords:** Dental public health, Oral diseases

## Abstract

**Introduction:**

The positive attitude and behaviour of dental students can be improved during their undergraduate studies and is considered an essential factor in promoting the oral self-care habits of their patients and society in general.

**Aim:**

This study was conducted to evaluate the oral health knowledge, attitudes and behaviours among undergraduate dental students at a private university, based on the year of undergraduate studies.

**Material and methods:**

A self-administered questionnaire based on the Hiroshima University-Dental Behavioural Inventory was distributed among 783 undergraduate dental students from 1st to 5th year. Statistical analysis was performed using SPSS version 20. The Mann–Whitney *U* test and one-way ANOVA tests (Kruskal–Wallis test) were used.

**Results:**

The scores of oral health knowledge, attitude and behaviour between preclinical and clinical dental students were found to have statistically significant differences (*P* < 0.001). The variation of knowledge, attitude and behaviour scores from 1st to 5th year undergraduate studies was shown to be statistically significant with the year of study (*P* *<* 0.001). The study showed significant improvement in the knowledge, attitude and behaviour of the final year dental students as compared with the 1st year dental students.

**Conclusion:**

Preclinical students need properly designed oral health educational programs to increase their attitude and behaviour toward oral health.

## Introduction

Attitude is defined as ‘the way in which a person views and evaluates something or someone. Attitudes determine whether people like or dislike things and therefore how they behave towards them’.^1^ Dental students are expected to play a critical role in instructing public oral health**;** their attitude reflects their understanding of the importance of disease prevention and their responsibility for improving their patients’ oral health.^2^ Therefore, the level of their own oral health behaviour can serve as positive models for their patients, families and friends.^3^

The Hiroshima University-Dental Behavioural Inventory (HU-DBI) questionnaire revealed interesting facts regarding differences in dental health attitude and behaviour among undergraduate dental students. So, it may pave the way for developing new programs aimed at improving dental students’ attitude and behaviour. Thus, the dental universities could assess their curricula to encourage needful oral health practices and positive behavioural attitudes. Thus, allowing them to modify their curricula at all levels of dental education.^4^

Many studies used the questionnaire to assess gender differences in oral health behaviours^5–7^ and variations between the cultures.^8,9^ Furthermore, Cortes et al.^10^ showed that oral health behaviour and attitudes improve with educational level.

Changing and modifying the dental curriculum will provide dental students with the knowledge, attitude and behaviour needed. This could also affect their oral self-care habits as well as increase their responsibility to motivate good oral health habits in their patients.^11–13^

There is limited data regarding the attitude and behaviour of dental students in Egypt. Hence, the purpose of this study is to assess the oral health knowledge, attitude and behaviour to answer our research question which is the difference of oral health knowledge, attitude and behaviour among undergraduate dental students from all levels.

## Materials and methods

### Study setting and participants

This cross-sectional study of oral health knowledge, attitude and behaviour of dental students in a dental school at a private University was carried out in the academic year 2015–2016 after approval from the Dental Research Ethics Committee of Faculty of Dentistry, Cairo University. A convenience consecutive sampling technique was employed to select dental students from 1st to 5th level who were invited to complete the questionnaire after their lectures. Only those students agreeing to participate in the study were considered. Arabic and English self-administered questionnaire, consisting of 20 items in a dichotomous response based on HU-DBI^14^ was distributed among 783 students. Preclinical students were included from 1st, 2nd and 3rd years. While, clinical students were included from 4th to 5th year.

To limit information bias, they were provided with a full explanation concerning the nature of the study, allowed to interact with the researcher for understanding the questions without leading their answers and asked to write exactly what they feel, conduct and perform to provide us with the best of their knowledge.

### Questionnaire

The questionnaire was designed to record self-reported oral health (knowledge) (items no: 2,8,10,15,19), oral health attitude (items No: 6,11,14) and oral hygiene behaviour (items no: 4,9,12,16).

Items 4, 9, 11, 12, 16 and 19 are given 1 point if the respondent agrees. Also, Items 2, 6, 8, 10, 14 and 15 are given 1 point if the respondent disagrees. Hence, the maximum possible score is out of 12 and the minimum score is 0. The higher the score means the better the oral health attitude and behaviour for each student. HU-DBI questionnaire included eight dummy items which are not included in the final scoring system.^15^

### Statistical analysis

The statistical analysis was performed using the software SPSS version 20. The difference of the oral health knowledge, attitude and behaviour between preclinical and clinical students was assessed by Mann–Whitney *U* test. The variation of the scores from 1st to 5th year students of each of knowledge, attitude and behaviour was analysed using Kruskal–Wallis test. *P* ≤ 0.05 was considered significant.

## Results

A total of 783 dental students, the HU-DBI inventory were completed by 780 students of which 372 were males and 408 were females. The participation was highest in the 1st year (179) and lowest in the 5th year (136) as shown in Table 1. The overall mean HU-DBI score was 6.77 ± 1.73.

**Table 1:** Academic levels, number of participants, mean and standard deviation of individual scores

Academic Yea	Total number	Total number of males	Total number of females	Participating students	Mean of individual scores	SD
Level 1	179	79	100	179	6.12	1.84
Level 2	152	75	77	152	6.05	1.31
Level 3	154	73	81	154	5.99	1.65
Level 4	162	80	82	159^a^	6.83	1.93
Level 5	136	66	70	136	6.77	1.73
Total	783	372	408	780	6.77	1.73

^a^Three excluded questionnaire

The percentages of “agree” responses to the 12 items of the HU-DBI questionnaire were presented in Table 2. Bleeding gums (item-2) were reported in 29.8% of the participants; 60.1% answered that it was impossible to prevent gum disease with only tooth brushing (item-14); and 56.1% reported that they postponed going to the dentist until they had a toothache (item-15). In addition, 39.9% of the students felt that they sometimes took too much time to brush their teeth (item-19). A higher response of “agree” for item-12 (“Checking the teeth in the mirror after brushing”; 82.3%), and item-9 (“brush each of my teeth carefully”; 65.6%) was found, reflecting higher aesthetic awareness among the dental students.

**Table 2:** HU-DBI questionnaire item, percentage of agree response and level of dental education [agree/(agree + disagree) × 100]

	Level 1	Level 2	Level 3	Level 4	Level 5	Total
	Total	Total	Total	Total	Total	
2— My gums tend to bleed when I brush my teeth. (D)**	43.0%	27.6 %	34.4 %	28.4 %	11.0 %	29.8 %
4— I have noticed some white sticky deposits on my teeth. (A)*	38.5 %	27.0 %	40.9 %	39.8%	40.7%	37.4 %
6— I think that I cannot help having false teeth when I am old. (D)	20.8 %	15.1%	14.3%	13.0 %	6.6%	14.3 %
8—I think my teeth are getting worse despite my daily brush. (D)	23.5 %	21.7 %	29.6 %	17.9 %	19.9 %	22.5%
9— I brush each of my teeth carefully. (A)	65.2%	64.5%	63.6%	71.6%	62.5%	65.6%
10— I have never been taught professionally how to brush. (D)	41.9 %	36.2 %	39.6 %	29.0 %	27.9 %	35.2 %
11— I think I can clean my teeth well without using toothpaste. (A)	19.7 %	13.2 %	14.9 %	30.8 %	27.9 %	21.2 %
12—I often check my teeth in a mirror after brushing alone. (A)	86.4 %	82.1 %	77.3 %	83.5 %	81.6 %	82.3 %
14— It is impossible to prevent gum disease with tooth brushing alone. (D)	55.6 %	68.4 %	59.7 %	58.5 %	58.8 %	60.1 %
15— I put off going to the dentist until I have a toothache. (D)	57.5 %	58.6 %	61.0 %	51.9 %	50.7 %	56.1 %
16— I have used a dye to see how clean my teeth are. (A)	6.7 %	5.3%	3.9%	8.2 %	5.9%	6.0%
19— I feel I sometimes take too much time to brush my teeth. (A)	39.1 %	40.8 %	38.3 %	45.9 %	34.6 %	39.9 %

^*^A = Agree, one point was given for each of the agree responses

**D = Disagree, one point was given for each of the disagree responses

Table 3 reveals the difference between each of the knowledge, attitude and behaviour scores between preclinical and clinical students analysed using Mann–Whitney *U* test. Clinical students’ scores were significantly higher than preclinical ones for knowledge, attitude and behaviour with *P* *=* 0.000, *P* *=* 0.000, *P* *=* 0.026, respectively.

**Table 3:** Comparison of the scoring between the preclinical and clinical students

	Knowledge		Attitude		Behaviour	
	Mean rank	*P* value	Mean rank	*P* value	Mean rank	*P* value
Preclinical year	359.77	0.000	367.78	0.000	377.56	0.026
Clinical year	441.03		427.85		411.77	

*P* ≤ 0.05 (statistically significant), *P* ≤ 0.001 (statistically highly significant)

Kruskal–Wallis test was carried out to compare the percentage of agree responses scores of knowledge, attitude and behaviour of 1st–5th year students. The test showed a statistically significant difference in knowledge, attitudes and behaviours among all levels with *P* = 0.000, *P* = 0.001, *P* = 0.026, respectively.

**Fig. 1:**
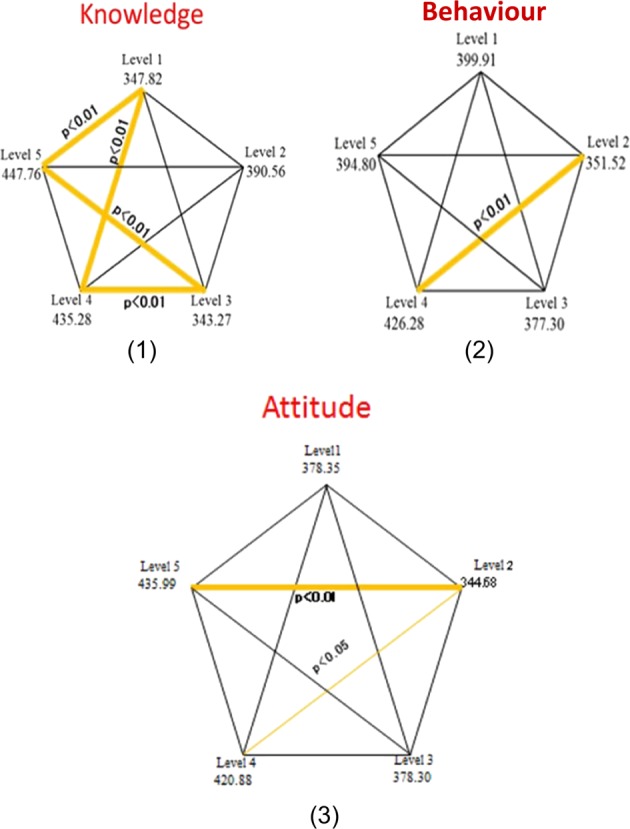
A Penta histogram of the knowledge, behaviour and attitude of all academic levels

Figure 1(1–3) show a Penta histogram for the knowledge, attitude and behaviour among all five academic levels. In Fig. 1(1), the yellow lines show that there was a statistically significant difference in attitude of students between year 2 and 4 with a *P* < 0.05, as well as between level 2 and 5 with a *P* < 0.01. In Fig. 1(2) there was a statistically significant difference in behaviour of students between year 2 and 4 with a *p* < 0.01.

In Fig. 1(3) the yellow lines show that there was a statistically significant difference in knowledge of students between level 1 and 4 with a *P* < 0.01 and level 1 and 5 with a *P* < 0.01, as well as there was a statistically significant difference between level 3 and 4 with a *P* < 0.01 and level 3 and 5 with a *P* < 0.01. While the black lines show no statistical significant difference.

## Discussion

Hiroshima University Dental Behaviour Inventory questionnaire (HU-DBI) is an efficient instrument in identifying differences in oral health behaviours among dental students from different countries due to the difference in the health education systems^16^ and the curriculum dissimilarities of dental universities.^8^ It could be used as a basis for developing additional programs aimed at improving dental students’ attitudes and behaviours. The dummy items were included in the original HU-DBI questionnaire to establish a rapport and help in gaining the students participation in the study.

The lowest agree responses reported for bleeding gum (item-2) was in level 5 compared with other levels. This might reflect their awareness towards periodontal disease prevention due to increase in their clinical experience. This finding is in accordance with the percentage of agree responses of level 5 in Patiala, India^17^ and approximately the same in Jordan.^18^

Dental students’ awareness about microbial dental plaque was reported in (item-4) if they noticed some white sticky deposits on their teeth. There was a low percentage in agree responses for all levels and it might be due to misinterpretation of this question as whether their teeth were clean or not instead of being aware of plaque.^13^ Also, during our questionnaire distribution, students were asking about the meaning of white sticky deposits.

A lower percentage of clinical students agreed that they “can’t help having false teeth when they were old” in (item-6) than preclinical students, this might be explained by fact that as educational level progressed, students become more attentive about their dentition, aware of the limitations and the impact of dentition loss on their dental function and aesthetic.^19^ This percentage is in accordance with that in UAE.^20^

In addition, most of the preclinical students had less oral health knowledge as they thought that their teeth would get worse despite daily brushing (item-8) and they had never been taught professionally how to brush their teeth (item-10). This can be explained by the fact that students had low oral health awareness and poor knowledge when they started their dental education. A possible cause of this is the lack of effective school-based oral health programs at the national level that seek to help children improve and maintain their oral health.^11^ Since the dental students are not receiving this before they enter dental school, oral health programs can be included in the preclinical curriculum to promote oral health awareness and knowledge. The finding of items 8 and 10 is in accordance to that in UAE^20^ and Britain.^9^

A higher total agree responses for (item-9) “brush each of my teeth carefully” and (item-12) “Checking the teeth in the mirror after brushing” were reported in all academic years, reflecting higher aesthetic awareness among the dental students. This percentage is very close to that in Bangalore, India.^21^

Most of the dental students agreed that it was impossible to prevent gum disease with tooth brushing alone (item-14), because of their belief that the most effective method to prevent dental caries and gum diseases is the teeth brushing,^11^ but there are other methods and techniques with brushing, they may be unaware of. This is similar to the total percentage of agree responses in UAE.^20^

Majority of dental students from all levels reported that they “put off going to the dentist until they have toothache” (item-15), which is similar to frequencies stated among dental students in Japan.^22^ This might be due to the high cost of dental services as well as fear of pain, previous bad dental experiences and the time required for frequent visits in agreement with the study done by Dagli et al.^23^

Regarding the total mean scores in this study, there was poor knowledge, attitude and behaviour among dental students and this might be attributed to that preventive courses, knowledge, experience of dental students gained from their basic dental subjects and training had minor influence on their own oral health attitude and behaviour. This is in accordance to the total mean score in Japan,^24^ India,^23^ Turkey^13^ and Croatia.^25^ This was higher than Chinese,^9^ Sudanese^6^ and Yemeni^7^ dental students, but lower than British^9^ and Japanese students.^22^ This discrepancy may be related to difference in the curriculum of the school, cultural attitude and behaviour.

The comparison of knowledge, attitude and behaviour between preclinical and clinical students revealed that knowledge, attitude and behaviour of clinical students are higher than that of preclinical ones. This could be due to the increasing experience of the clinical students about oral health care being in contact with patients in clinical environment. In addition, as they progress in their dental education, students may become more conscious of their overall health and more attentive to oral health related issues; therefore, they adopt better oral health attitude and behaviour. This finding is in consistence with previous study done in Turkey, Lithuania and India by^13,26,27^, respectively who found that oral health attitude and behaviour of clinical students is higher than that of preclinical. In contrast, Al-shiekh et al.^6^ found no differences in the oral health attitude and behaviour of clinical students compared with preclinical students in Sudan. This may be attributed to the weak knowledge of students to preventive dentistry and this is reinforced by the fact that the dental students who participated in the study were taught preventive dentistry during year 4 according to the University’s curricula and this might have caused a poor effect on their attitude and behaviour.

This study revealed that oral health knowledge, attitude and behaviour improved with increasing academic levels, indicating that there is increase in the dental education experience gained from basic dental subjects, preventive courses and clinical training. This result is consistent with the results reported in a study comparing HU-DBI questionnaire between Greek and Japan^22^ and other studies in Jordan,^18^ in Nigeria^28^ and in India^17^ as they showed that oral health attitude and behaviour improved by educational levels. By contrast, results reported by Halboub et al.^7^ revealed that oral health attitude and behaviour of dental students in Yemen were not improved with increasing levels of education that means faulty understanding for the acquired knowledge and their translation into negative attitude and behaviour which necessitates critical revision of the curricula.

This study was carried out in only one private dental school and thus it might limit the generalizability of the results all over Egypt. So, more studies are needed in different dental school in Egypt. Using the HU-DBI in this study reported interesting facts regarding differences in oral health attitudes and behaviours between undergraduate dental students. Dental students, as future health professionals, should have a comprehensive dental educational oral health in school programs including self-care regimes and preventive courses starting from the 1st year of dental education.

## Conclusions

Within the limitations of this study, we conclude the following:

1. The oral health knowledge, attitude and behaviour of preclinical dental students were lower than that of clinical ones.
2. Preclinical students need properly designed oral health educational programs to increase their attitude and behaviour toward oral health.
